# Improving Non-Destructive Concrete Strength Tests Using Support Vector Machines

**DOI:** 10.3390/ma8105368

**Published:** 2015-10-22

**Authors:** Yi-Fan Shih, Yu-Ren Wang, Kuo-Liang Lin, Chin-Wen Chen

**Affiliations:** 1 Department of Civil Engineering, National Kaohsiung University of Applied Sciences, Kaohsiung 80778, Taiwan; shih090202@kimo.com (Y.-F.S.); jessica199111@gmail.com (C.-W.C.); 2 Faculty of Civil and Ecological Engineering Department, I-Shou University, Kaohsiung 84001, Taiwan; kllin@isu.edu.tw

**Keywords:** concrete compressive strength, non-destructive test, rebound hammer test, ultrasonic pulse velocity test, artificial intelligence, support vector machines

## Abstract

Non-destructive testing (NDT) methods are important alternatives when destructive tests are not feasible to examine the *in situ* concrete properties without damaging the structure. The rebound hammer test and the ultrasonic pulse velocity test are two popular NDT methods to examine the properties of concrete. The rebound of the hammer depends on the hardness of the test specimen and ultrasonic pulse travelling speed is related to density, uniformity, and homogeneity of the specimen. Both of these two methods have been adopted to estimate the concrete compressive strength. Statistical analysis has been implemented to establish the relationship between hammer rebound values/ultrasonic pulse velocities and concrete compressive strength. However, the estimated results can be unreliable. As a result, this research proposes an Artificial Intelligence model using support vector machines (SVMs) for the estimation. Data from 95 cylinder concrete samples are collected to develop and validate the model. The results show that combined NDT methods (also known as SonReb method) yield better estimations than single NDT methods. The results also show that the SVMs model is more accurate than the statistical regression model.

## 1. Introduction

To determine the actual compressive strength of cast concrete, destructive tests are the most reliable methods. Typically, test samples are taken from the mixed concrete batch on site and then sent to the laboratory for curing and testing. Then, destructive tests are conducted to obtain the actual concrete compressive strength. Nevertheless, the concrete compressive strength test results in the laboratory might not be representative for the *in situ* cast concrete because factors such as concrete transportation, placement, tamping, and curing would affect the concrete quality and strength. In light of this, one alternative is to take core samples directly from the finished concrete structure and conduct destructive compressive tests on these drilled cylinder samples to determine the concrete strength. However, it is not always possible to take core samples on site and the structure might be damaged during the drilling process. As a result, non-destructive test (NDT) might be a good alternative to measure concrete strength at times when destructive tests are not preferable.

Two popular non-destructive test methods to estimate concrete compressive strength are the rebound hammer test (RH) and the ultrasonic pulse velocity test (UPV). For the rebound hammer test, a spring-controlled mass strikes the surface of the test object and the rebound is measured. The rebound of the mass depends on the hardness of the surface it strikes and the rebound values can be related to the compressive strength of the concrete [1]. For the ultrasonic pulse velocity tests, two transducers are set against two surfaces of the test object. Ultrasonic waves are sent from one transducer and received by the other during the test. The travelling time of the ultrasonic pulse to pass from one surface to the other is recorded. With a known distance between the two surfaces of the test object, the velocity of the ultrasonic wave can be obtained. Generally speaking, the speed is higher when the concrete quality is better, in terms of density, uniformity, and homogeneity. As a result, the ultrasonic pulse velocity can be related to concrete compressive strength as well [2].

Although non-destructive test methods are more economical and time-efficient comparing to destructive tests, one major drawback is that they are not very reliable when the test results are used to estimate the concrete compressive strength. For rebound hammer tests, it is shown that concrete compressive strength estimations have an average of over 20% mean absolute percentage error when comparing to the “actual” compressive strength obtained by destructive tests [3]. Combined methods (combining rebound hammer tests and ultrasonic pulse velocity tests, also known as SonReb) are proposed by researchers to improve the concrete compressive strength estimations and positive results are obtained [4,5,6,7,8,9].

Most of these previous researches attempted to relate the concrete compressive strength with rebound hammer values and ultrasonic pulse velocity using statistical regression (linear or non-linear) analysis. This research proposes a novel regression approach by incorporating an artificial intelligence method (support vector machines; SVMs) as an alternative regression approach. Research has shown that SVMs have better regression ability when comparing to traditional statistical regression analysis [10,11,12] and, hence, this research intends to incorporate SVMs to improve the regression results. In addition, more sample data are collected to improve the reliability of the research results. For most of the previous researches, only 10 to 20 samples are collected; in contrast, this research collected data from a total of 95 test samples. In order to collect these test samples, the research team cooperated with a certified construction material testing laboratory and obtained both destructive and non-destructive test results from 95 concrete cylinder samples. The data collected are used to develop and test the SVMs concrete compressive strength prediction model. The hammer rebound values and ultrasonic pulse velocities are set as the SVMs model input and the actual compressive strengths from destructive tests are set as the model output. Finally, results from SVMs model are compared with the results from traditional statistical regression analysis.

## 2. Non-Destructive Testing Methods

Two popular non-destructive testing methods for measuring concrete compressive strength (rebound hammer tests and ultrasonic pulse velocity tests) are adopted in this research and they are briefly introduced in the following two sections.

### 2.1. Rebound Hammer Test

**Figure 1:**
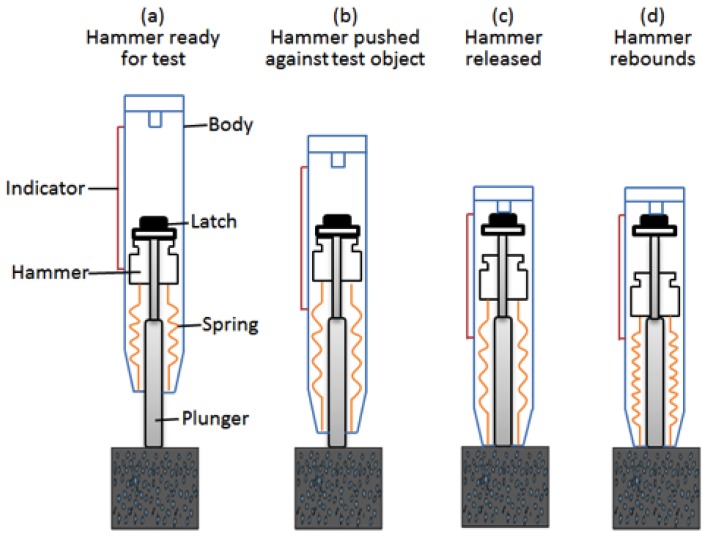
Rebound hammer test process.

As one of the most popular non-destructive tests for measuring concrete compressive strength, the rebound hammer test provides a convenient and relatively inexpensive way to estimate the concrete compressive strength. The rebound hammer test is based on the principles that the rebound of an elastic mass depends on the hardness of the surface the mass strikes. The extent of the rebound is an indication of the surface hardness of the test object. In the case of the concrete compressive strength test, low strength and low stiffness concrete will yield a lower rebound value due to more energy absorption [13]. During the rebound hammer test, the plunger of the rebound hammer is first pressed against the surface of the concrete, and then a gradual increase in pressure is applied until the hammer impacts. The rebound value is read from a graduated scale and is designated as the rebound number or rebound index (*Q*-value). The concrete compressive strength can be estimated using the conversion table provided by the manufacturer [14]. Figure 1 illustrates this process.

The research team cooperated with a local certified material testing laboratory for the data collection. By regulation, contractors are required to prepare test samples on site during the concrete casting and then send these samples to a certified laboratory for compressive strength tests. After initial casting, the concrete cylinder samples are demolded in the laboratory in 48 ± 4 h. After demolding, the samples are immediately put in the moist cabinet for curing under calcareous water at a constant temperature of 23.5 degree Celsius. For this research, all the concrete cylinder samples are prepared according to Chinese National Standards. The test samples are kept in the moist cabinet for 28 days after initial casting and then are taken out for both non-destructive and destructive tests. It should be noted that the destructive tests are conducted using an HT-8391 200T computer-controlled concrete compression test machine (Hung Ta Instrument Co., Ltd., Taichung, Taiwan) to obtain the actual concrete compressive strength.

**Figure 2:**
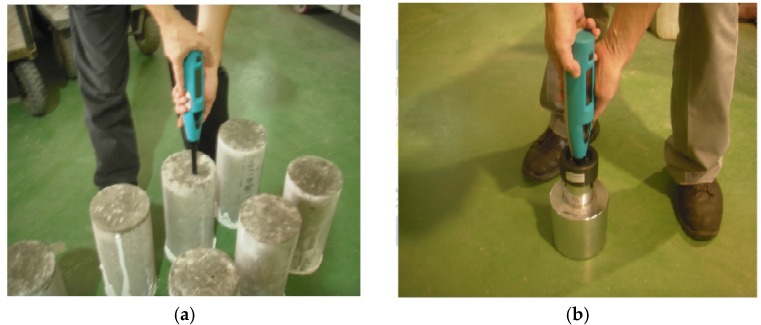
(**a**) Rebound hammer test; (**b**) rebound hammer calibration.

For the rebound hammer test, the experiment is conducted according to ASTM C805 specifications by a trained technician. Ten rebound hammer tests are conducted, evenly distributed on top of each circular concrete cylinder sample (12 cm × 24 cm), as shown in Figure 2a. If any one reading is seven units away from the overall average, that particular reading is disregarded. If there are two readings seven units away from the overall average, this set of 10 readings is disregarded. From April to June 2015, a total of 95 concrete samples are collected for this research. It should be noted that the rebound hammer is calibrated each day before the tests are started, as shown in Figure 2b.

### 2.2. Ultrasonic Pulse Velocity Test

**Figure 3:**
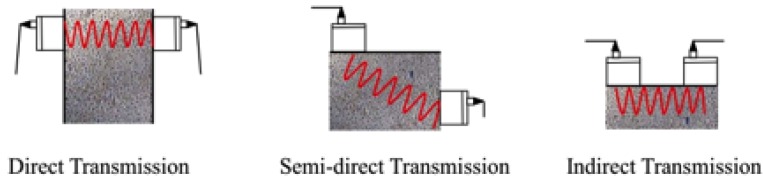
Ultrasonic pulse velocity test methods.

Since the 1940s, the pulse velocity of longitudinal stress waves was applied to effectively measure the transmission velocity in the concrete [1]. This test method is applicable to assess the uniformity and relative quality of concrete, to indicate the presence of voids and cracks, and to evaluate the effectiveness of crack repairs [15]. It is also applicable to indicate changes in the properties of concrete, and in the survey of structures, to estimate the severity of deterioration or cracking. The ultrasonic pulse velocity method measures the travel time of an ultrasonic pulse (50–54 kHz) passing through the concrete. Comparatively higher velocity is obtained when concrete quality is good in terms of density, uniformity, homogeneity, *etc*. The tests begin when an ultrasonic pulse is generated and transmitted from an electro-acoustic transducer, placed in contact with one surface of the concrete. After passing through the concrete, the vibrations are received and converted by the electro-acoustic transducer at the other end of the surface. The elapsed time between input and output of the wave is measured with precision of at least 0.1 μs. With known travelling distance, *D*, and the travel time measured, *T*, the pulse velocity (*V* = *D*/*T*) can be calculated [16,17]. According to different settings of the transducer locations, there are three types of ultrasonic pulse velocity tests, as shown in Figure 3.

**Figure 4:**
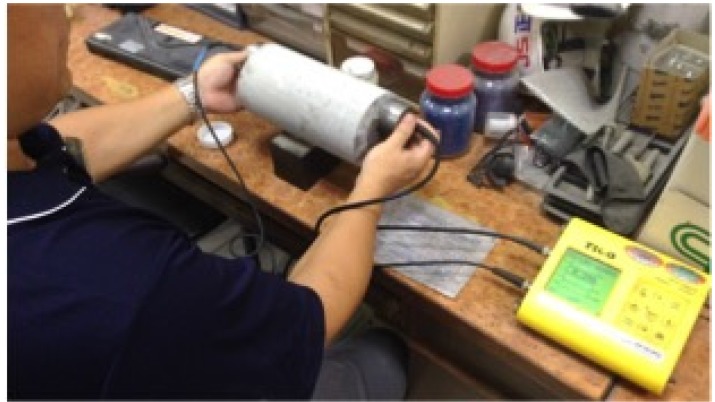
Ultrasonic pulse velocity test.

For this research, the ultrasonic pulse velocity test (direct transmission method) is conducted by a trained technician in the laboratory. Four measurements are taken for each of the circular concrete cylinder samples (12 cm × 24 cm), as illustrated in Figure 4. Each day before the tests, the instrument is calibrated to make sure the test results are consistent. Both rebound hammer tests and ultrasonic pulse velocity tests are conducted for each of the 95 test samples.

## 3. Support Vector Machines and Model Development

The support vector machines, SVMs, are a machine learning technique based on the minimization of structural risk and statistical learning theory and was first purposed by Vapnik [18]. Through nonlinear mapping into high-dimensional feature space, the SVMs are able to classify sample data using linear models. Similar to neural networks, the SVMs also involve training and testing of data instances. The SVMs try to minimize the upper bound of the generalization error and this enables the SVMs to have better generalizability even when dealing with unseen data. Compared to other artificial intelligence techniques, the SVMs have several advantages, such as efficient use of high-dimensional feature space, uniquely solvable optimization problems, and the ability to be theoretically analyzed using computational learning theory [19,20]. The concepts of the SVMs model are briefly described below [10].

Given a dataset $\text{G}=\left\{\left(x_{i},d_{i}\right)\right\}$, where *x_i_* is the input vector, *d_i_* is the target value, and *n* is the size of the dataset, through a non-linear mapping (Φ) of *x* into the high-dimensional feature space, the non-linear regression in the low-dimensional space can be represented by the linear regression in the high-dimensional space. It can be expressed as:

(1) f(x)=ωΦ(x)+b

where ω is the weight vector; Φ is the high-dimensional feature space, and *b* is the bias of the hyper plane.

The principles of the SVMs are to minimize the structural risks. As a result, ω and *b* can be obtained by minimizing the risk penalty function below:

(2) $$R_{SVR}\left(C\right)=C\times \frac{1}{n}\sum_{i-1}^{n}L\text{ε}\left(d_{i},y_{i}\right)+\frac{1}{2}{\left\|\text{ω}\right\|}^{2}$$

where

(3) $$L\text{ε}=\{\begin{array}{ccc} \left|d-y\right|-\text{ε} & & if\left|d-y\right|\geq \text{ε}\\ 0 & & otherwise \end{array}$$

In Equation (2), $C\times \frac{1}{n}\sum_{i-1}^{n}L\text{ε}\left(d_{i},y_{i}\right)$ is the estimated risk based on *L*ε (ε—insensitive Loss Function). $\frac{1}{2}{\left\|\text{ω}\right\|}^{2}$ is the penalty item for estimating the structural risk. *C* is the penalty constant and can be used to control the level of penalty when error occurs.

In order to estimate ω and *b*, slack variables ξ and ξ^*^ are introduced and the new objective function becomes:

Minimize

(4) $$R_{SVMs}\left(\text{ω},{\text{ξ}}^{\left(*\right)}\right)=C\times \frac{1}{n}\sum_{i-1}^{n}\left({\text{ξ}}_{i}+{\text{ξ}}_{i}^{*}\right)+\frac{1}{2}{\left\|\text{ω}\right\|}^{2}$$

subject to:

(5) $$\begin{array}{c} d_{i}-\text{ωφ}(x_{i})-b_{i}\leq \text{ε}+{\text{ξ}}_{i}\\ \text{ωφ}(x_{i})+b_{i}-d_{i}\leq \text{ε}+{\text{ξ}}_{i}^{*} \end{array}\begin{array}{cc} & {\text{ξ}}^{\left(*\right)}\geq 0 \end{array}$$

By introducing the Lagrange multipliers, *a_i_* and a_i_^*^, the SVMs decision function becomes:

(6) $$f\left(x,a_{i},a_{i}^{*}\right)=\sum_{i=1}^{n}\left(a_{i}-a_{i}^{*}\right)K\left(x,x_{i}\right)+b$$

Next, the Lagrange multipliers are introduced into the penalty objective function and the dual function can be obtained as:

Maximize

(7) $$R\left(a_{i},a_{i}^{*}\right)=\sum_{i=1}^{n}d_{i}\left(a_{i}-a_{i}^{*}\right)-\varepsilon \left(a_{i}+a_{i}^{*}\right)-\frac{1}{2}\sum_{i=1}^{n}\sum_{j=1}^{n}\left(a_{i}-a_{i}^{*}\right)\left(a_{j}-a_{j}^{*}\right)K\left(x,x_{i}\right)$$

subject to:

(8) $$\sum_{i=1}^{n}\left(a_{i}-a_{i}^{*}\right)=0,\begin{array}{c} 0\leq a_{i}\leq C\begin{array}{cc} & i=1,2,\ldots ,n \end{array}\\ 0\leq a_{i}^{*}\leq C\begin{array}{cc} & i=1,2,\ldots ,n \end{array} \end{array}$$

*K*(*x_i_*,*x_j_*) is defined as the kernel Function, which is the inner product of *x_i_* and *x_j_* in the corresponding feature spaces ψ(*x_i_*) and ψ(*x_j_*), $K\left(x_{i},x_{j}\right)=\text{φ}(x_{i})*\text{φ}(x_{j})$. Kernel functions are functions that satisfy Mercer’s conditions and there are four common types of kernel functions used in SVMs: Linear Kernel, Polynomial Kernel, Sigmoid Kernel, and Radial Basis Function (RBF) Kernel. Among them, RBF is favorable for its capability of dealing with nonlinearity and high-dimensional computation, and effectiveness in reducing complexity for inputs by adjusting *C* and γ [21]. For this reason, this research uses the RBF Kernel when building the SVMs classifiers. The RBF Kernel is illustrated in Equation (9):

(9) $$K(x_{i},x_{j})=\exp\left(-\text{γ}{(x_{i}-x_{j})}^{2}\right)$$

where γ is a constant.

For this research, the SVMs model development is implemented in the Matlab environment using the LS-SVMlab software developed by Suykens *et al.* [22]. The least square support vector machine, LSSVM, is first introduced by Suykens *et al.* [23]. The LSSVM is chosen because it eases the heavy computation burden for the constrained optimization programming for the SVMs by introducing equality instead of inequality constraints. In addition, LSSVM utilizes a least squares loss function instead of the ε—insensitive loss function. In this way, the problem is simplified in such a way that the solution is characterized by a Karush-Kuhn-Tucker (KKT) linear system instead of a computationally hard quadratic programming (QP) problem [24,25].

**Figure 5:**
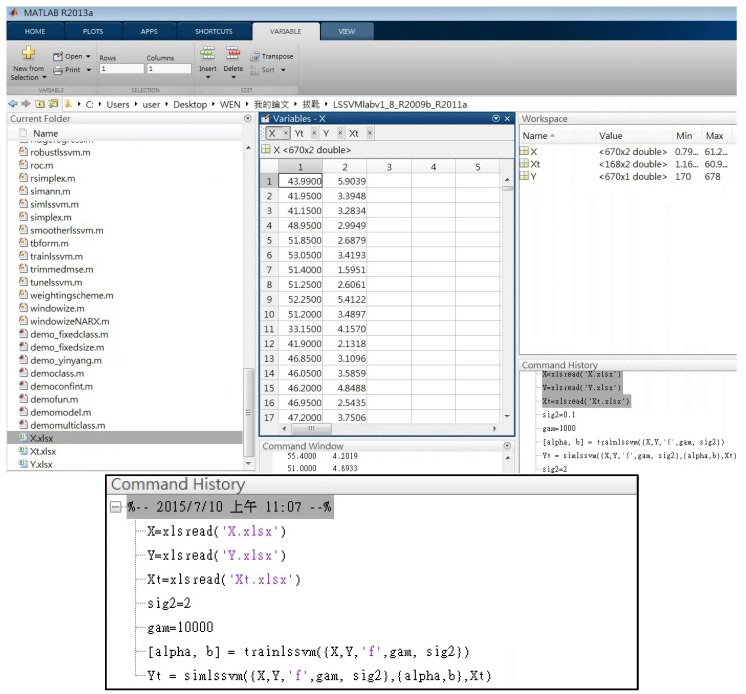
SVMs model development process and commands in Matlab.

The hammer rebound (*Q*-values) and ultrasonic pulse velocities measured from the test samples are taken as the model inputs and the actual compressive strengths are set as the model outputs. Among the 95 obtained test samples, 85 of them are randomly chosen as the training dataset and the remaining 10 samples are set as testing dataset for model validation. Both the training and testing datasets (EXCEL files) are first loaded into the Matlab environment and then used for the model development and testing. The Matlab environment and command window for the model development and testing process is illustrated in Figure 5. The 85 sample data in the training dataset are used to train the SVMs model using the “trainlssvm” command in the Matlab. Afterwards, the trained SVMs are called up to generate predictions using the input data from the testing dataset (using “simlssvm” command). Then, the model prediction outputs are compared with the actual compressive strengths in the testing dataset to examine the prediction accuracy. It should be noted that the parameters *C* and γ for the RBF kernel need to be determined during the SVMs model development. While a larger *C* indicates a stronger smoothing, a higher value of γ forces the model to fit the training data more closely [21]. There is no particular set of rules to determine these parameters and the optimal set of parameters is obtained by minimizing the training prediction bias through a series of trial and error in this research. For this particular sample, the parameters *C* and γ obtained for the best SonReb SVMs model developed in this research are 2 and 10,000 respectively.

## 4. Results Analysis

To improve the non-destructive test for estimating the concrete compressive strength, SVMs models are developed for the combined NDTs (SonReb) using both the results from rebound hammer and ultrasonic pulse velocity tests as the model inputs. As the SVMs are supervised learning algorithms, the actual compressive strengths from destructive compression tests are set as the target output to train the model. Among the 95 collected test samples, 85 of them are randomly selected as the training dataset and the remaining 10 are set as the testing dataset. The ten samples in the testing dataset are “unseen” or new data for the trained SVMs model and they can be used to test the prediction ability of the model.

The model prediction accuracy is measured by the mean absolute percentage error (MAPE) illustrated in the equation below:

(10) $$MAPE=\frac{1}{n}\sum_{i=1}^{n}\left|\frac{A_{i}-P_{i}}{A_{i}}\right|$$

where *A_i_* is the actual concrete sample compressive strength and *P_i_* is the model predicted value.

For comparison purpose, a total of three SVMs models are developed with different input variable settings: (a) Hammer rebound value only; (b) Ultrasonic pulse velocity only; and (c) Hammer rebound value + ultrasonic pulse velocity. The output variable is set as the actual compressive strength for all three models. After training with the 85 test sample data, the 10 samples in the testing dataset are used to examine the model prediction accuracy. The model prediction outputs (estimated concrete compressive strength) are compared with the actual compressive strengths obtained by destructive compression tests. The model prediction results are summarized in Table 1.

**Table 1:** SVMs model prediction accuracy.

SVMs Models With Different Input Variable	Mean Absolute Percentage Error (MAPE) (%)
(a) Rebound Hammer (RH) Test	8.92%
(b) Ultrasonic Pulse Velocity (UPV) Test	9.25%
(c) SonReb (RH+UPV) Test	6.77%

As shown in Table 1, the SVMs model with two input variables (hammer rebound value + ultrasonic pulse velocity) yields the best prediction results (6.77% MAPE). The MAPEs are obtained by comparing the model predicted concrete compressive strength with actual concrete compressive strength. As for the remaining two models with only one input variable, rebound hammer test model yields better prediction results (8.92% MAPE) when comparing to ultrasonic pulse velocity test model results (9.25% MAPE).

For comparison, linear regression with two input variables (hammer rebound value + ultrasonic pulse velocity) is also conducted. To be consistent, the same 85 training data are used to develop the multi-variable linear regression model. The linear equation obtained is illustrated in Equation (11). With the obtained linear equation, data from 10 testing dataset are used to calculate the concrete compressive strength estimation. The prediction results are summarized in Table 2 and the MAPE for the multi-variable linear regression is 8.73%.

*Y* = 1.25733*R* + 0.0182*V* − 65.387 (11)

where *Y* is model output (compressive strength, N/mm^2^); *R* is hammer rebound value and *V* is ultrasonic pulse velocity.

When comparing the overall prediction accuracy, the MAPE for the SVMs model is 6.77% and the MAPE for the linear regression model is 8.73%. From Table 2, it can be observed that most of the individual estimations from the SVMs are closer to the actual compressive strength. In addition, the variations are smaller for the SVMs model when the estimation errors are examined. Nevertheless, it should be noted that results obtained from this analysis are specific for the sample data collected in this research. It is a broad indication that the SonReb method produces better estimation than the single test method. Also, SVMs can be a good alternative to linear regression when developing the prediction model.

**Table 2:** SVMs and linear regression model prediction.

Concrete Compressive Strength (N/mm^2^) and Mean Absolute Percentage Error (MAPE,%)				
SVMs Model		Linear Regression Model		Actual Strength
Strength	MAPE	Strength	MAPE	Strength
28.44	3.94%	29.00	6.01%	27.36
28.99	12.54%	28.14	15.11%	33.15
29.52	12.23%	25.33	24.68%	33.64
26.63	9.93%	25.47	5.16%	24.22
28.03	13.86%	26.27	6.72%	24.61
26.64	3.68%	25.22	8.80%	27.65
27.83	3.58%	29.78	10.82%	26.87
26.71	5.17%	27.14	6.85%	25.40
26.85	0.80%	26.68	1.42%	27.07
27.49	1.94%	27.44	1.75%	26.97

## 5. Conclusions

In order to improve the non-destructive test (NDT) for concrete compressive strength estimation, an artificial intelligence-based approach (support vector machines; SVMs) is proposed for the combined NDT method (SonReb), which consists of rebound hammer and ultrasonic pulse velocity tests. A local certified material testing laboratory is chosen for collaboration to conduct the tests and collect data for this research. Both non-destructive tests (rebound hammer and ultrasonic pulse velocity tests) and destructive tests (compression tests) are conducted on circular concrete cylinder samples (12 cm × 24 cm). Data from a total of 95 test samples are collected to develop and test SVMs prediction models. Among the 95 test samples, 85 of them are randomly selected as the training dataset for the proposed SVMs model development. The remaining ten test samples are grouped as the testing dataset for SVMs model validation. A total of three SVMs models are developed with different input variable settings: (a) Hammer rebound value only; (b) Ultrasonic pulse velocity only; and (c) Hammer rebound value + ultrasonic pulse velocity. Actual concrete compressive strengths obtained from the destructive tests are the desired output for the proposed models. In order to compare the accuracy of the compressive strength estimations, the mean absolute percentage error (MAPE) is adopted as the accuracy measure in this research. The results show that the combined NDT (SonReb) SVMs model yields the best prediction result (6.77% MAPE) when comparing to the predictions from the other two models with single input variable (hammer rebound values only or ultrasonic pulse velocities only). The MAPEs for these single-input-variable SVMs models are 8.92% and 9.25%, respectively. In addition, the prediction results from multi-variable regression analysis are also obtained for combined NDT method (SonReb) and the corresponding MAPE is 8.73%. The research results have shown that combined NDT (SonReb) tests yield better estimation results and the SVMs model improves the non-destructive test estimation for concrete compressive strength.
